# Fiber Sensor Systems Based on Fiber Laser and Microwave Photonic Technologies

**DOI:** 10.3390/s120505395

**Published:** 2012-04-27

**Authors:** Hongyan Fu, Daru Chen, Zhiping Cai

**Affiliations:** 1 Department of Electrical Engineering, Xiamen University, Xiamen 361005, China; E-Mails: fuhongyan@xmu.edu.cn (H.F.); zpcai@xmu.edu.cn (Z.C.); 2 Institute of Information Optics, Zhejiang Normal University, Jinhua 321004, China

**Keywords:** fiber optics sensors, sensor interrogators, fiber lasers, microwave photonics, microwave photonic filters, microwave photonic generation, fiber Bragg gratings

## Abstract

Fiber-optic sensors, especially fiber Bragg grating (FBG) sensors are very attractive due to their numerous advantages over traditional sensors, such as light weight, high sensitivity, cost-effectiveness, immunity to electromagnetic interference, ease of multiplexing and so on. Therefore, fiber-optic sensors have been intensively studied during the last several decades. Nowadays, with the development of novel fiber technology, more and more newly invented fiber technologies bring better and superior performance to fiber-optic sensing networks. In this paper, the applications of some advanced photonic technologies including fiber lasers and microwave photonic technologies for fiber sensing applications are reviewed. FBG interrogations based on several kinds of fiber lasers, especially the novel Fourier domain mode locking fiber laser, have been introduced; for the application of microwave photonic technology, examples of microwave photonic filtering utilized as a FBG sensing interrogator and microwave signal generation acting as a transversal loading sensor have been given. Both theoretical analysis and experimental demonstrations have been carried out. The comparison of these advanced photonic technologies for the applications of fiber sensing is carried out and important issues related to the applications have been addressed and the suitable and potential application examples have also been discussed in this paper.

## 1. Introduction

Fiber optics sensors have been a very attractive technology due to their overwhelming advantages in industrial applications, such as compactness, lightweight, high sensitivity, immunity to electromagnetic interference, the ease of multiplexing and so on. Fiber optics sensors can be applied to measure a large variety of parameters, e.g., temperature, strain, refractive index, bending, loading, liquid level, *etc.* The study of fiber optics sensors started no later than 1977 [1–4], and since the nnumerous novel types of fiber optics sensors have been studied and demonstrated. So far, some kinds of fiber optics sensors have already been commercialized for industry applications, e.g., FBGs have been successfully applied for structure health monitoring (SHM) of large-scale civil infrastructures like bridges, dams and tunnels, and performance monitoring of key equipment and machinery and so on [5].Technologies for sensing networks, including interrogation and multiplexing techniques have also been studied intensively, aiming for high channel count, fast system response and better long term performance with low cost, e.g., spectrally coded multiplexing techniques [5], fiber loop ring down cavity sensing systems [6], *etc.* Nowadays, with the dramatically increasing research on fiber technology, the research on some advanced photonic technologies such as fiber lasers and microwave photonics technology has started to help realize novel fiber optic sensing applications.

Fiber lasers [7,8] have attracted great attention due to their wide applications in areas such as optical fiber sensors, optical fiber communications, free space optical communications, optical signal processing, medical equipment, large infrastructure construction, the manufacturing industry, defense industry and so on. Compared with the dye laser, the gas laser, the semiconductor laser, and the conventional solid state laser, fiber lasers show advantages such as fiber compatibility, low threshold, high efficiency, high quality of the laser beam, large wavelength range, flexibility in wavelength control, and reliability in a harsh environment. The history of the fiber laser is almost the same as the laser. Early in 1961, Snitzer developed the optical fiber amplifier and the fiber laser. Lasing in glass fibers was achieved in 1960 [9–11]. Low loss erbium-doped fibers were developed in the 1980s [12], which was the basis of the development of the erbium-doped fiber amplifier and erbium-doped fiber lasers. Besides the rare earth doped optical fiber, the semiconductor laser which was developed in 1970 provided a powerful pump for the fiber laser. Several different types of the fiber lasers were developed in the 1990s. Fiber laser-based nonlinear optics was also proposed, which greatly enlarged the wavelength range of the fiber lasers. In 1990, Kashyap used the FBG in the fiber laser to enhance the mode selection [13]. The first distributed Bragg reflector (DBR) fiber laser by writing the FBG in 0.5-m erbium-doped fiber was demonstrated with a single-mode operation [14]. In 1993, Mizrahi fabricated the DBR fiber laser with a 2.5-cm cavity [15]. In the 21st century, the performance of fiber lasers were further improved, many commercial fiber lasers appeared and the applications of the fiber laser greatly increased. Fiber laser based sensors and sensor systems are also developed. Multi-wavelength fiber lasers [16,17], mode-locking fiber lasers [18,19] and other new fiber lasers [20–22] are used in sensing network systems.

Microwave photonics is a newly developed technology, which by definition is an interdisciplinary research area that studies the interaction between microwave and light wave signals, and the photonic devices, subsystem sor optoelectronic devices operating at the microwave frequencies [23–25]. Microwave photonic technology enjoys the advantages of large bandwidth, low loss, immunity to electromagnetic interference, the capability of wireless communications, *etc*. Therefore, since the 1990s, microwave photonics technology and its applications for fiber wireless communication systems, radar systems and signal processing and generating has been studied extensively, among which photonic generation and processing of microwave signals, optically controlled phased array antennas, radio-over-fiber systems *etc.* are several active research topics in this area. Photonic generation of microwave signals of up to 40 GHz by using optical four wave mixing and fiber Bragg gratings has been reported [26]; photonic generation by using dual-wavelength fiber ring lasers has also been proposed and demonstrated [27–31]. Different types of microwave photonic filters have also been studied intensively [32–34]. A photonic band pass filter with high skirt selectivity and stopband attenuation has been proposed in [35,36] by using an ovel dual-cavity band pass optical structure based on two pairs of active fiber Bragg grating cavities, which formed an infinite impulse response (IIR) filter. A finite impulse response (FIR) microwave photonic filter with a rejection up to 60dBimplemented by slicing light after a superstructured FBG and dispersive medium was achieved [37]. Microwave photonic filters with negative coefficients have also been developed [38–41]. With the development of microwave photonics technology, the applications of this technology have started to attract research attention and the application of microwave photonic technology in communication and sensing systems has been exploited [42–44].

In this review, we will provide a comprehensive overview of the applications of advance photonic technology, including fiber lasers and microwave photonics for sensing systems, and the newest and most promising techniques for sensing applications. The remainder of this review article will be divided into three parts: in the first part, the fiber laser technology and its applications in sensing systems will be reviewed; in the second part, the microwave photonic technology and its applications in sensing systems will be reviewed; finally come the conclusions.

## 2. Fiber Lasers and Their Sensing Applications

### 2.1. Fiber Lasers Sensing Applications

**Figure 1.**
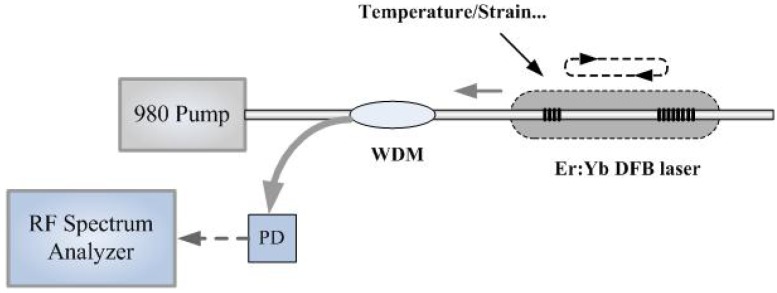
The diagram of the dual polarization DBR fiber laser based fiber sensor.

Fiber lasers are being widely used in sensor systems, in which they act as the optical light source or the sensor element. When the fiber laser is used as the sensor, it can usually achieve high signal-to-noise ratio (SNR), high sensitivity, long distance sensing and multi-parameter sensing. In 1993, Ball *et al.* reported the operation of an active, single-frequency, polarimetric, Bragg-grating fiber-laser strain sensor [45]. The short Bragg-grating fiber-laser has two orthogonal polarization modes which yielded a single beat frequency after optical mixing and the beat frequency provided the sensing information with a rate of −4.1 MHz/mstrain for linear strain and –0.37 MHz/(deg/cm) for torsional strain. Hadeler *et al.* reported the application of a dual polarization distributed feedback (DFB) fiber laser which was used as a strain and temperature sensor [46]. By measurement of the absolute wavelength of one polarization as well as the polarization beat frequency, strain and temperature were determined simultaneously. The demonstrated sensor has an accuracy of ±3 με and ±0.04 °C. Guan *et al.* demonstrated a novel fiber-optic hydrophone that uses a dual polarization DBR fiber laser as the sensing element, whose experimental scheme is shown in Figure 1 [47].

The operation principle of the sensor is based on the modulation of the birefringence of the fiber laser by high-frequency ultrasound. The amplitude and frequency of the acoustic pressure, and temperature can be determined simultaneously by measuring the amplitude and frequency of the sidebands as well as the polarization beat frequency of the output of the fiber laser using a photo-detector and a radio-frequency spectrum analyzer. The DBR fiber laser hydrophone has a linear response to acoustic pressure and can detect acoustic frequency up to at least 40 MHz. Han proposed a simple and flexible multiwavelength Raman-fiber-laser-based remote-sensor for simultaneous measurement of strain and temperature by use of FBGs [48]. By combining two uniform FBGs with a tunable chirped fiber grating, simultaneous two-channel sensing probes with a high extinction ratio of more than ∼50 dB over a 50-km distance is achieved. Since the two uniform FBGs have identical material composition and different cladding diameters, this allows simultaneous measurement of strain and temperature for long-distance sensing applications over more than 50 km. Radio-frequency (RF) beat frequencies between two longitudinal modes and two polarization modes of a birefringent dual-longitudinal-mode moiré DFB fiber laser are also proposed [49] to measure strain and temperature simultaneously with accuracy of ±15 με and ±0.2 °C.

### 2.2. Fiber Laser-Based FBG-Interrogation

Fiber Bragg grating (FBG), one of the most promising types of fiber sensors, has attracted great attention due to its advantages of small size, fiber compatibility, immunity to electromagnetic interference, and multi-point sensing capability [50]. Multi-FBG sensing is one of the most attractive techniques to achieve a sensing network, since it can exhibit the advantages of FBG sensing, achieve quasi-distributed multi-point sensing, and reduce the cost of each FBG sensor. In the FBG sensing network, multi-FBG interrogation is the most challenging technique. In principle, time-division multiplexing (TDM) and wavelength-division multiplexing (WDM) are the two basic techniques in a multi-FBG sensing system. There are several typical methods for multi-FBG interrogation, such as by employing scanning Fabry-Pérot filters [51], matched grating filters [52] and arrayed waveguide grating filters [53] to detect the optical signal reflected by multiple FBGs from a broadband optical source or broadband fiber lasers. These passive sensing systems not only lead to a low SNR due to poor efficiency in the use of the optical source, but also cannot distinguish identical FBGs at different locations along the fiber. By utilizing the fiber laser, recently developed active FBG sensing systems [17, 22,54] have been demonstrated with advantages such as a high SNR and the capability for remote sensing.

**Figure 2.**
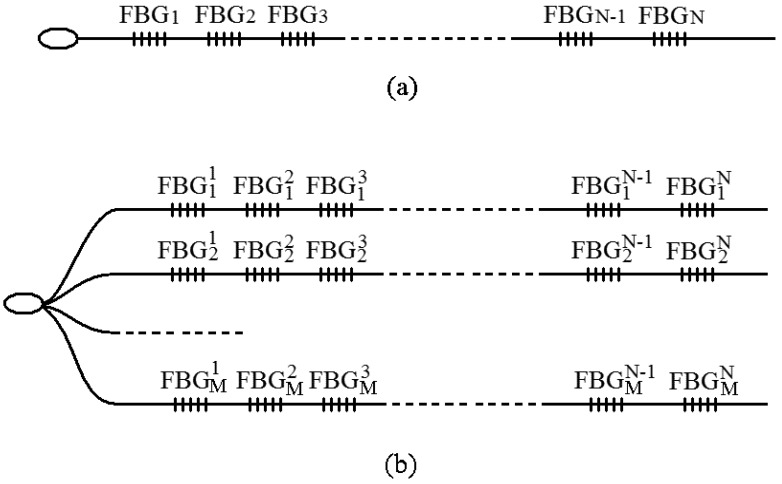
Network topology of the FBG sensing system. (**a**) Single line structure; (**b**) Multi-line structure.

For an FBG-based sensing network system, single line structures or multi-line structures, which are shown in Figure 2, areoften used for the network topology.

A typical TDM technique for multi-FBG interrogation was proposed based on a passively mode-locked fiber laser in 1997 [18]. It is well known that a passively mode-locked fiber laser source has a relatively large bandwidth in the wavelength domain which permits interrogation of many FBG arrays and a relatively short pulse in the time domain which is good to distinguish FBGs at different locations along the fiber. The authors used a passively mode-locked erbium-doped fiber laser with an output pulse of tens of picoseconds' duration and output optical bandwidth of 85 nm (FWHM). The laser output is launched into 3.25 km of DCF and then into the FBG array to be interrogated. Reflections from individual FBGs propagate back through the dispersive fiber and are monitored by a fast detector and a sampling oscilloscope. The high dispersion of the DCF converts strain- and temperature-induced wavelength shifts into a shift in the pulse arrival time at the detector. Temporally, the reflected signal from an array of FBGs is thus a sequence of pulses separated by the time of flight between the gratings, plus a wavelength-dependent delay resulting from the double-pass through the DCF. In 2004, Peng proposed an intensity and wavelength-division multiplexing FBG sensor system using a tunable multiport fiber ring laser [17]. FBGs are arranged in multi-line fiber structure with each line connected to one output port of the fiber ring laser. Different output ports of the fiber ring laser have different output power, so even identical FBG can be used in this sensor system when they are used in different output port. A Fabry-Pérot filter is introduced in the fiber ring laser which ensures multi-FBG interrogation based on the WDM technique. The multiplexing number of the sensing gratings is limited by the reflective band of each FBG sensor and the whole bandwidth of the light source. There are also some reports for the FBG sensor system by combining WDM and TDM techniques such as the hybrid wavelength-time-domain interrogation system for multiplexed fiber grating sensors based on a strain-tuned erbium-doped fiber laser [55].

### 2.3. FDML Fiber Laser-Based FBG-Sensing Network

A novel fiber laser, Fourier domain mode locking (FDML) fiber laser was recently proposed for optical coherence tomography applications [20]. The FDML is analogous to active laser mode locking for short pulse generation, except that the spectrum rather than the amplitude of the light field is modulated. High-speed, narrowband optical frequency sweeps are generated with a repetition period equal to the fundamental or a harmonic of cavity roundtrip time and a strain of highly chirped, very long optical pulses can be achieved. We recently proposed an FBG-interrogation application based on the FDML fiber laser [21] and further improved the active sensing network based on FBGs and the FDML fiber laser [22].

A passive FBG-sensing network based on the FDML fiber is introduced here. By converting wavelength to time measurement, FBG interrogation for a sensing system based on a continuous-wave FDML fiber laser is demonstrated, which possesses of advantages of low cost, potentially high speed and multi-point sensing based on universal FBGs.

**Figure 3.**
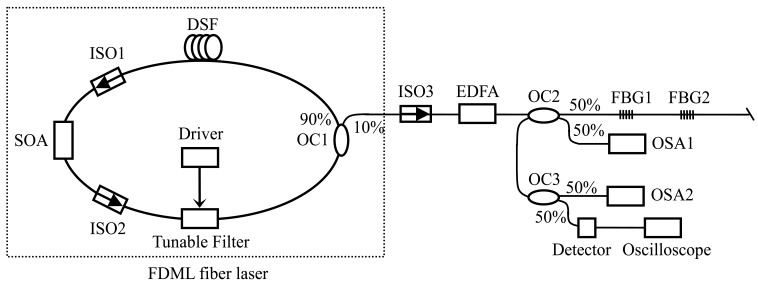
Experimental setup of the proposed FBG sensing system based on a FDML fiber laser.

Figure 3 shows the experimental setup of the FBG sensing system. An FDML fiber laser is shown in the dotted box, which consists of two isolators (ISO1 and ISO2), a semiconductor optical amplifier (SOA), a section of dispersion-shifted-fiber (DSF), a fiber Fabry-Pérot filter (FFP-TF), and an optical coupler (OC1) with the 10%-ratio port used as the output port of the laser.

The length, insert loss, zero dispersion wavelength, and dispersion slope of the DSF are about 11.5 km, 3 dB, 1,549 nm, and 0.085 ps/km/nm^2^, respectively. The 3-dB bandwidth of the electrically tunable filter with an operation wavelength range from 1,520 nm to 1,570 nm is 0.1 nm. A programmable function generator (HM8130) is used as a driver of the tunable filter. A C-band erbium-doped fiber amplifier (EDFA) is used to amplify the output of the FDML fiber laser. Two FBGs with central wavelengths of 1,548.9 nm and 1,546.6 nm, and reflectivity of 96.8% and 95.2% (for FBG1 and FBG2, respectively) are used as sensors. Two 3-dB optical couplers (OC2 and OC3) are used to ensure the measurement of the output of the FDML fiber laser and the sensing signal. An optical spectrum analyzer (OSA1 or OSA2, AQ6317) is used to measure the optical spectra of laser output and sensing signal. A lightwave detector (HP83440D) and an oscilloscope (DSO6052A) are used to measure the sensing signal in time domain.

**Figure 4.**
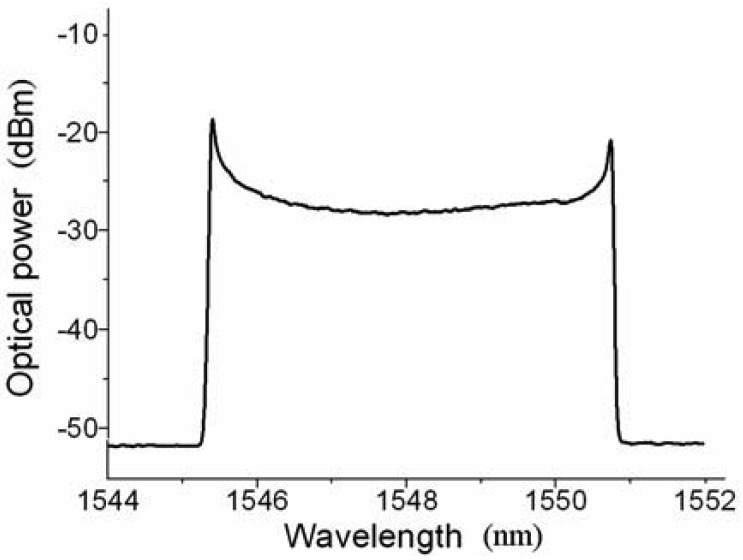
Experimentally measured optical spectrum of the FDML fiber laser.

The programmable function generator with sinusoidal waveform output is used to drive the tunable filter in our experiment. When the frequency of the driver signal is adjusted to be 19.464 kHz, 38.921 kHz, and 58.366 kHz, respectively, FDML operations of the fiber laser are achieved. Note that the frequency of the driver signal is only limited by the mechanical response of the tunable filter. Figure 4 shows the optical spectrum of the amplified output of the fiber laser when the frequency, the bias voltage, and the amplitude of the driver signal are 19.464 kHz, 1.0 V, and 730 mV, respectively. The output spectrum width and the central wavelength are dominated by the amplitude (∼7.5 nm/V) and the bias voltage of the dirver signal, which indicates the the number of the FBG sensors in this system is only limited by the gain band of the SOA and the operation wavelength range of the tunable filter. Since the central wavelength has not been tuned outside the gain band of the SOA, the output of the FDML fiber is continuous wave with different wavelength at different time.

**Figure 5.**
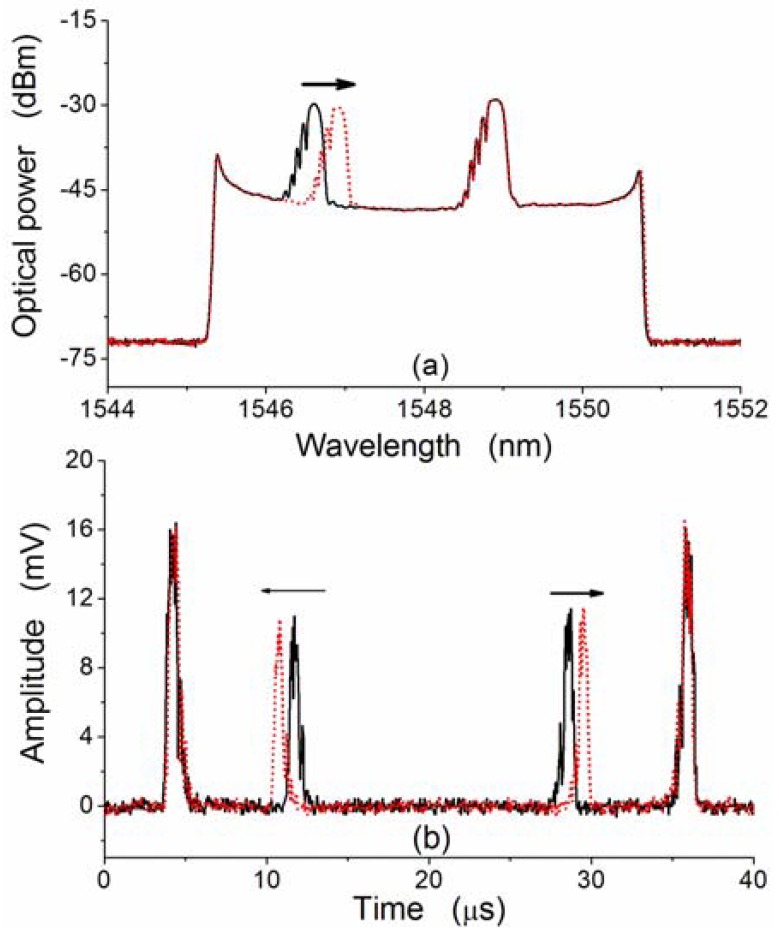
(**a**) Optical spectrum and (**b**) time-domain spectrum of the reflected light of the two FBGs.

Two FBGs are used to pick up part of the output of the FDML fiber laser at the central wavelengths of the two FBGs. Accordingly, in time domain two FBGs just pick up part of the output of the FDML fiber laser at certain time, resulting in pulse signals in time domain. The pulse location in time domain is determined by the central wavelength of the FBG when the output spectrum of the FDML fiber laser is fixed. Figure 5(a) shows the experimentally measured optical spectra of the two-FBG sensing signal when the two FBGs are in original state (black curve) or the FBG2 is tuned under strain (red dotted curve). Accordingly, a strain of pulses can be observed in time domain and Figure 5(b) shows the experimentally measured time-domain spectrum of the two-FBG sensing signal (within one cavity round-trip time) when the two FBGs are in original state (black curve) or the FBG2 is tuned under strain (red dotted curve). Within one cavity round-trip time, the central wavelength of the tunable filter match the central wavelength of one FBG twice, which results in two pulses within one cavity round-trip time. The time shift of pulse (about 0.895 μs) correspondes to the wavelength shift of the central wavelength of FBG2 (about 0.313 nm). Thus, converting wavelength to time measurement is achieved for the FBG interrogation. One should note that the ratio between the wavelength shift of the central wavelength of FBGs and the time shift of the pulses depends on both the original wavelength position in the output spectrum of the FDML fiber laser (since a sinusoidal waveform output of the driver is used) and spectrum bandwidth of the output of the FDML.

The above experiments have shown the possibility to utilize the FDML fiber laser for FBG-interrogation. However, it is still a passive sensing system where the FDML fiber laser acts as a broadband light source.We further proposed a novel multi-FBG sensing system based on a spectrum-limited (SL-) FDML fiber laser, whose output spectrum is determined by the FBGs. The mode locking operation of the SL-FDML fiber laser provides a unique method to determine the spatial positions of the FBGs. Both wavelength- and spatial-domain interrogations for multiple FBGs are achieved. The proposed SL-FDML fiber laser with a fast wavelength-swept operation (up to the order of 100 kHz) ensures high-speed FBG interrogation and offers potential applications for dynamic signal sensing. In addition, the proposed active FBG sensing system based on the SL-FDML fiber laser can also be used for remote sensing.

**Figure 6.**
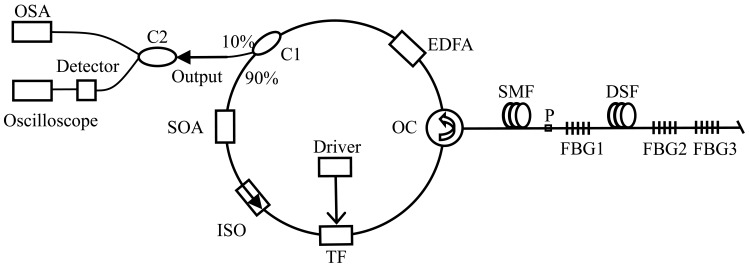
Schematic illustration of the FBG sensing system based on a SL-FDML fiber laser.

Figure 6 shows the schematic configuration of the proposed FBG sensing system based on a SL-FDML fiber laser. Different from the FDML fiber laser in Figure 2, both an EDFA and a SOA are used as the gain media of the fiber laser. Three FBGs are arranged in series and are used as both the sensors in the system and the wavelength-selected components in the laser. They are connected to the cavity by a 2.6-km single mode fiber (SMF) and a 0.8-km dispersion-shifted fiber (DSF). Note that there is a ferrule connector/physical connector (FC/PC) connector point between SMF and FBG1. The central wavelengths and reflectivities of the three FBGs (FBG1, FBG2 and FBG3) are 1,547.8 nm, 1,548.9 nm, 1,546.7 nm and 92.5%, 96.8%, 92.1%, respectively. The measurements of the SL-FDML laser and the sensing signals are obtained with the use of a 3-dB optical coupler (C2). An OSA is used to measure the optical spectrum of the fiber laser. A lightwave detector (HP83440D) and an oscilloscope (DSO6052A) are used to measure the sensing signal in the time domain.

**Figure 7.**
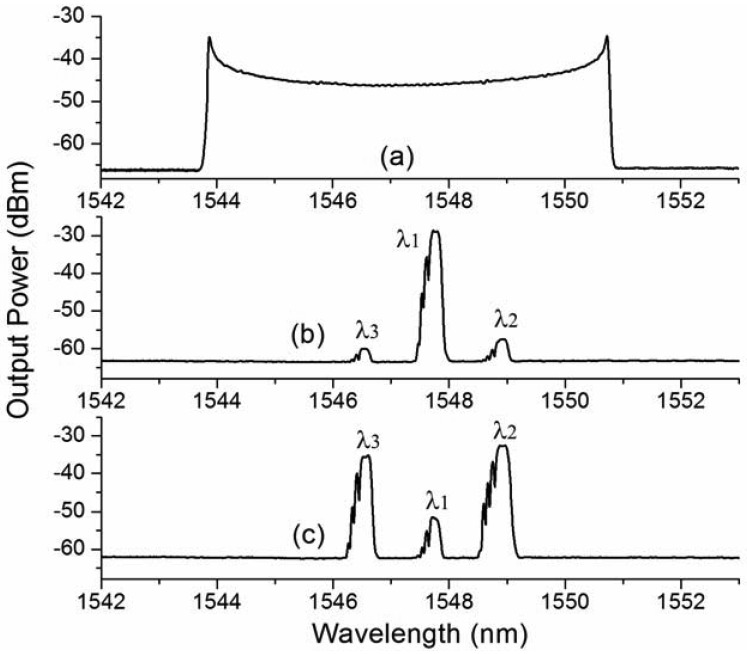
Output spectra of (**a**) the FDML fiber laser, (**b**) the SL-FDML fiber laser driven at 37.432 kHz, and (**c**) the SL-FDML fiber laser driven at 28.776 kHz.

**Figure 8.**
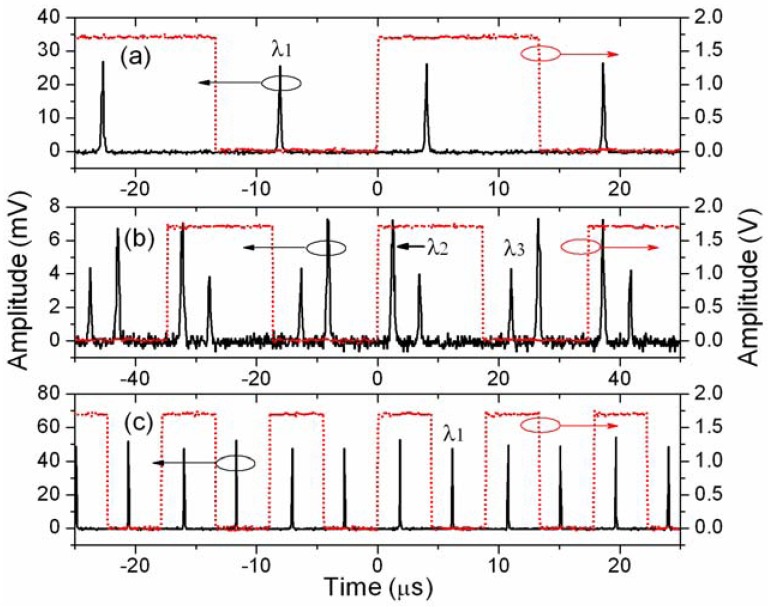
Time-domain spectra (solid curves) of the SL-FDML laser when the driving frequency is (**a**) 37.432 kHz, (**b**) 28.776 kHz, and (**c**) 112.30 kHz. Dotted curves show the trigger signal of the driver.

When we disconnect the P point (to introduce a Fresnel reflection of 4%), conventional FDML operation is achieved when a sine wave frequency of 37.444 kHz is utilized to drive the tunable filter. The frequency agrees well with the fundamental frequency of the fiber cavity with a measured length of about 5.4 km. Figure 7(a) shows the output spectrum of the FDML fiber laser with Fresnel reflection feedback. In a SL-FDML fiber laser, FBGs are used to select the wavelength of the laser and to generate pulsed output. Figure 7(b,c) shows the output spectra of the SL-FDML fiber laser when the driving frequencies for the tunable filter are 37.432 kHz and 28.776 kHz, respectively. The frequencies match with the calculated fundamental frequencies of the cavities defined with FBG1 and FBG2/FBG3. Figure 8 shows the time-domain spectra (solid curves) of the SL-FDML fiber laser when the driving frequency is (a) 37.432 kHz, (b) 28.776 kHz, and (c) 112.30 kHz (three times the cavity round-trip frequency with FBG1 feedback). Dotted curves show the trigger signal of the driver. Here we can summarize some characteristics of the SL-FDML fiber laser: the output spectrum is determined by the FBGs; the lasing wavelengths can be chosen by adjusting the driving frequency of the tunable filter; two pulses appear within one driving period and the pulse position in time is determined by the center wavelength of the FBG; lasing at two or more wavelengths is possible once the FBGs are arranged close enough to each other (for example, FBG2 and FBG3 have a spacing of about 0.8 m).

**Figure 9.**
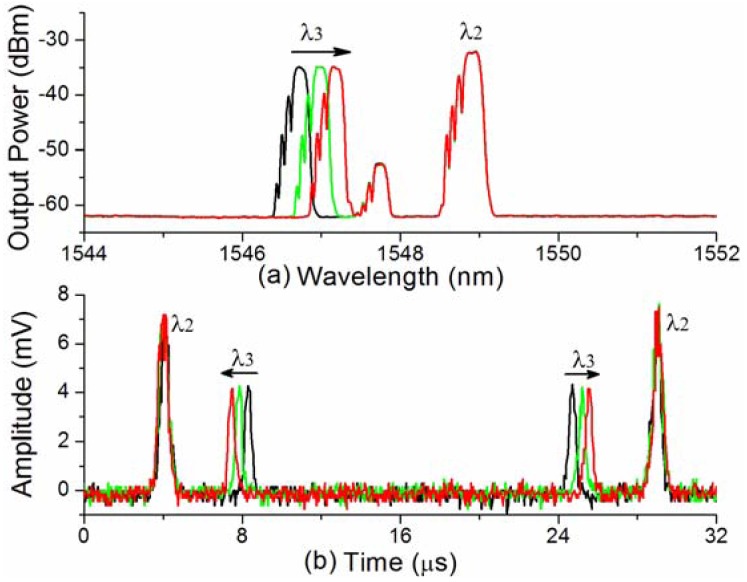
(**a**) Experimentally measured optical spectrum and (**b**) time-domain spectrum of the SL-FDML fiber laser when FBG3 is under strain tuning.

This novel SL-FDML fiber offers an efficient mothed method for the interrogation of multiple FBGs. Figure 9(a) shows the experimentally measured optical spectrum and Figure 9(b) depicts the corresponding time-domain spectrum (b) of the SL-FDML fiber laser containing FBG2 and FBG3 when FBG3 is tuned under strain.The ratio between the time shift of the pulses and the shift of the center wavelength of FBG3 is about 0.55 μs/nm, andis dependent on the original wavelength of the FBG, the scanning range and driving frequency of the tunable filter. Note that the ratio will remain a constant for a given driving signal if a triangular wave is used to drive the tunable filter.

**Figure 10.**
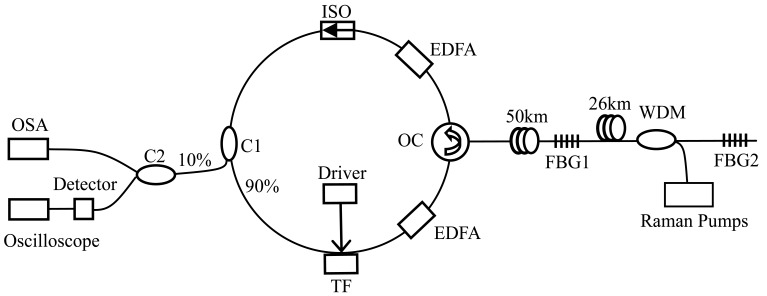
Setup of the ultra-long-distance FBG sensor system based on an SL-FDML fiber laser.

The SL-FDML fiber laser also shows the capability to achieve long distance sensing. A novel ultra-long-distance (up to 76 km) FBG sensor system based on a SL-FDML fiber laser is proposed [56]. Figure 10 shows the setup of the ultra-long-distance FBG sensor system based on a SL-FDML fiber laser combining a Raman amplifier to compensate the transmission loss of the long fiber. Strain sensing based on such an ultra-long distance FBG sensor system is demonstrated with a signal-to-noise ratio over 22 dB and a sensitivity of 1.03 pm/με in the wavelength domain or 18.3 ns/με in the time domain.

The three properties, fast wavelength sweeping, mode locking, the mapping of the wavelength domain and the time domain, make the FDML fiber laser a good candidate for multi-FBG interrogation. The fast wavelength sweeping of the FDML fiber laser permits a dynamically measurement of some relatively slow signals. The mode locking property of the FDML fiber laser makes it possible to achieve location information of the FBGs. The mapping of the wavelength domain and the time domain in the FDML fiber laser can provide a relatively low-cost detection of the wavelength shift of the FBG. However, there are still some problems should be overcome before the practical application of the sensing system based on the FDML fiber laser. Firstly, the fiber Fabry-Pérot filter driven by a signal generator should be stabilized since its shift in wavelength will result in the pulse shift in time domain corresponding to the sensing FBG. A possible method is to stabilize and FBG (or other wavelength filter) to calibrate the fiber Fabry-Pérot filter. Secondly, although the mode locking property of the FDML fiber laser can provide achieve the FBG location information, it will also result in possible difficulty of location design of the FBGs and relatively longtime to achieve all FBG-interrogations. FBG-interrogation. In conclusion, the novel FDML fiber laser provides a new solution of the FBG sensing network and further research work should be done for its practical applications.

## 3. Microwave Photonics and Their Sensing Applications

### 3.1. Microwave Photonic Filters

**Figure 11.**
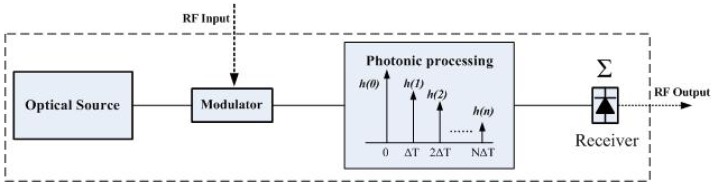
The structure of a typical time delay based microwave photonic filter.

A microwave photonic filter is a photonic subsystem that performs filtering functions at microwave frequencies. The scheme of a typical microwave photonic filter is shown in Figure 11. The microwave signal to be processed modulates the optical signal from the optical source via an optical modulator (it can be a Mach-Zehnder modulator, phase modulator or electro-absorption modulator), and then launches it into the photonic link composed of photonic devices, e.g., wavelength selective elements, attenuators, amplifier, fiber Bragg gratings and delay lines *etc.*, which can perform various functions like tapping, light weighting, and time delays and so on in the optical domain. Then the signal after photonic processing is sent to the receiver (photo-detector) to obtain the processed microwave signals.

Generally, the transfer function of this kind of incoherent microwave photonic filter with multiple taps can be expressed as Equation (1) [33]:

(1) $$H(\Omega )=\sum_{n=-\infty }^{\infty }h(n)e^{-jn\Omega \Delta T}$$

where, *h(n)* is the coefficient of the *n*th tap, Δ *T* is the time delay between taps. By designing the parameters *h(n)* and time delay between each tap, microwave photonic filter with different frequency responses can be achieved. Normally, microwave photonic filters have a periodical frequency response, and their free spectrum range (FSR) is decided by the time delay between taps, *FSR* = 1/Δ*T*, and time delay Δ*T* can be introduced by the real time delay, or by the dispersion induced time delay [33]. Microwave photonic filters based on a fiber Mach-Zehnder interferometer (FMZI, as a slicing filter) and dispersive media exhibits desirable single passband frequency response which is resulted from the sinusoidal and continuous optical sample weight distribution[57–59].

Microwave photonic filter can also be realized by directly filtering, and in [60] microwave filtering is realized by direct filtering of the sideband of modulated optical signal with designed FBGs. As the filtering characteristics of this kind microwave photonic filters are mainly limited by the optical devices which perform the direct filtering, such as bandwidth, and stability, *etc.*, this kind of filter is not easy to implement, especially since it's difficult to realize filters with ultra-narrow bandwidth.

### 3.2. Photonic Generation of Microwave Signals

**Figure 12.**
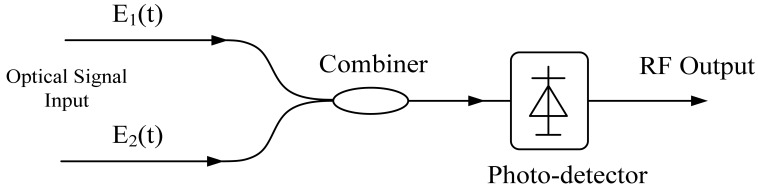
The schematic diagram for microwave generation by beating two different wavelengths.

Photonic methods are desirable for generating high frequency microwave signals. There have been two categories of microwave generating techniques by photonic methods. The first category is to beat the light of two different wavelengths to achieve microwave signals with the frequency equal to the wavelength spacing, whose principle is shown in Figure 12. Assuming that two optical waves *E*_1_ and *E*_2_, and their electronic fields are *E*_1_(*ω*)=|*E*_1_|exp[*j*(2*πω*_1_*t*+*ø*_1_)] and *E*_2_(*ω*)=|*E*_2_|exp[*j*(2*πω*_2_*t*+*ø*_2_)], so the intensity of the beating signal on the PD is expressed as Equation (2), which shows that the beating signal has the frequency equals to the frequency difference of the two optical waves, *i.e.*, ω = ω_1_ – ω_2_:

(2) $$\begin{array}{ll} I_{ph}={\left|E(\omega )\right|}^{2} & ={\left|E_{1}+E_{2}\right|}^{2}={\left|E_{1}\right|}^{2}+{\left|E_{2}\right|}^{2}+2\left|E_{1}\right|\left|E_{2}\right|\cos[2\pi (\omega_{1}-\omega_{2})t+(\phi_{1}-\phi_{2})]\\ & =P_{1}+P_{2}+2\sqrt{P_{1}P_{2}}\cos[2\pi \;ft+(\phi_{1}-\phi_{2})] \end{array}$$

A number of techniques have been proposed to realize the microwave generation by beating two wavelengths, such as optical injection locking technology to achieve highly coherent phases of two individual optical waves [61]; beating of dual wavelength fiber lasers in which since optical waves with two different wavelengths are generated from the same lasing cavity, their phases are correlated [25]; beating the higher order sidebands of the modulated light to get the higher frequency microwave multiplication signals, in which an RF signal with relatively low frequency is modulated onto an optical carrier (by a Mach-Zehnder Modulator or phased modulator), and several harmonic sidebands are generated, by filtering the sidebands with desirable frequency difference with optical filters, microwave signal with higher frequency can be generated [26,62,63], *etc*.

**Figure 13.**
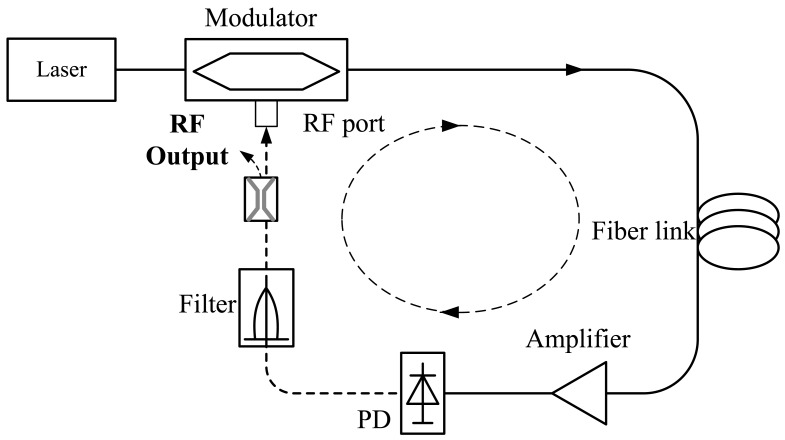
The schematic diagram of an optoelectronic microwave oscillator.

Another category for microwave signal generation is the optoelectronic microwave oscillator [64–68], by which microwave signals with much purer spectrum and higher quality can be generated, and the generating schematic diagram is shown as Figure 13. The only limitation of this generation technique is the bandwidth of the modulator and the electronic filters.

With the development of microwave photonic technology, the realization of microwave photonic signal generation and processing has become more and more convenient and cost effective, which in turn offers a very desirable choice for fiber sensing and sensing networks, especially for the harsh areas, due to its capability for handling fast signal processing speeds. In the following sections, we will give some examples of the applications of microwave photonic technologies in fiber sensing.

### 3.3. Microwave Photonic Filters for FBG Interrogation

**Figure 14.**
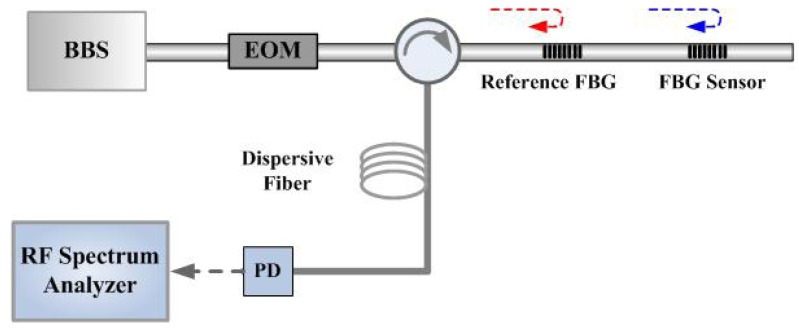
The operation diagram of the intensity-modulated fiber Bragg grating sensor based on RF signal measurement.

The microwave photonic filter can be adopted as an interrogator for the FBG sensing network. Dong *et al.* have proposed a FBG sensing interrogation system by using a reference FBG and a length of dispersive fiber [42], whose operation scheme is shown in Figure 14. The RF signal is measured after a photodetector, generated by two modulated optical signals reflected from the sensing FBG and a reference FBG. The wavelength shift of the sensing FBG will change the phase difference between the two optical signals as shown in Equation (3), and thus changesthe intensityof the RF signal. The temperature effect will be compensated by the reference FBG as the two FBG experience the same temperature changes and will shift at the same time:

(3) Δϕ=2πf(2nΔL/c+DLΔλ)

**Figure 15.**
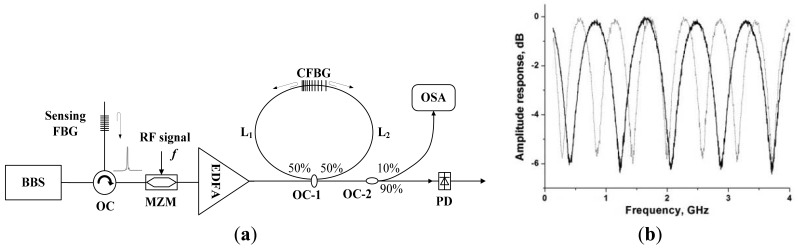
(**a**)The experimental setup of the sensing interrogator based on Sagnac loop microwave photonic filtering and (**b**) the measured frequency responses of the Sagnac loop based microwave photonic filter.

We have proposed a FBG sensing interrogation system using Sagnac loop-based microwave photonic filtering [69], whose experimental setup is shown in Figure 15(a). The output of this structure is a typical response of microwave photonic notch filter, whose frequency response can be expressed as:

(4) $$P_{e}=P_{0}[1+m\cos(\Delta \phi /2)\sin(2\pi \;ft+\phi_{0})]$$

where *P_0_* is the optical power reflected by the sensing FBG; *m, f* and *ϕ*_0_ are the modulation index of the MZM, the modulation frequency, and the phase of the output electrical signal from PD, respectively. The term Δ*ϕ* = 4*πf·n_eff_*·(*L*_1_−*L*_2_+2*z*−*l*)*/c* is the phase difference of the modulated signal experienced in two arms of the Sagnac-loop, and *L*_1_ and *L*_2_ are the lengths of two arms; *n_eff_, c*, and *l* are the refractive index of the fiber, speed of light in vacuum and the length of LCFBG, respectively; *z* is the position in the LCFBG where the light is reflected, and it is decided by the Bragg wavelength of the sensing FBG. The measured frequency responses of this filter are shown in Figure 15(b), when the length of two arms of the Sagnac loop is different.

**Figure 16.**
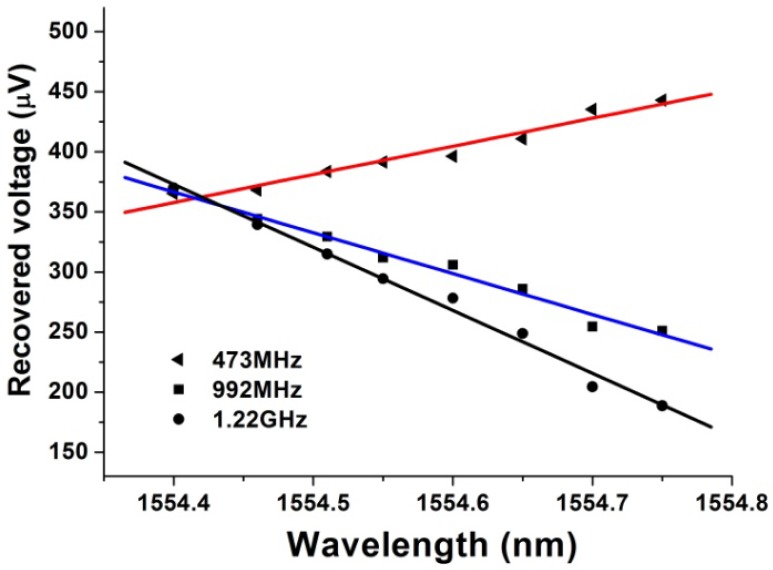
Relationship between recovered RF intensity and the wavelength of sensing FBG at different modulation frequencies (473 MHz: triangles; 995 MHz: squares; 1.22 GHz: circles).

When the wavelength of the sensing FBG changes with the variation of the measurands, the reflection position of its wavelength at the LCFBG changes, thus providing the transfer function of the microwave photonic filter shift. Since we modulated an RF signal with a fixed frequency onto the optical signal, the amplitude of the recovered RF signal changes with the wavelength shifts of the sensing FBG. The responsive factor of this sensing interrogation system is related to the modulation frequency. The experiment results of this interrogation system are shown in Figure 16, which gives the relationship between the recovered RF signal intensity and the wavelength of the sensing FBG at different modulation RF frequencies.

**Figure 17.**
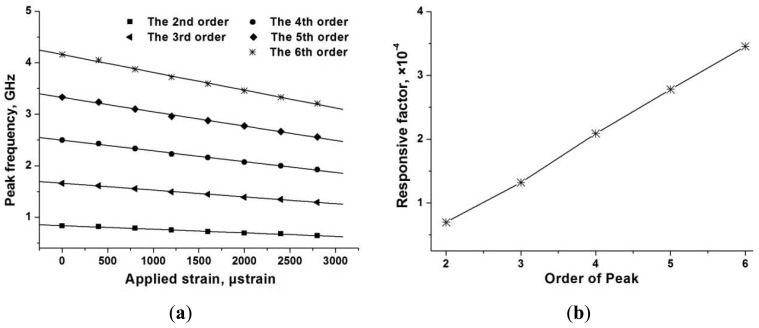
(**a**) The measured relationship between the frequency of different orders of peaks and the applied strain on the sensing FBG; and (**b**)the system sensing responsivity and the peak order.

One can also track the peak frequency for sensing interrogation. The measured relationship between the frequency of different order peaks and the applied strain can be illustrated in Figure 17(a), which shows that the sensing responsivity of the system is higher when we track a higher order peak. The measured relationship between the sensing responsivity and the peak order is shown in Figure 17(b).

This FBG sensing interrogation system can also measure the dynamic variation of the sensing FBG, and for this interrogation system, the issues below should be carefully considered for the system design and optimization:

1. The bandwidth and the reflectivity of the sensing FBG should be optimized, and it's better to utilize a sensing FBG with narrow bandwidth and high reflectivity;
2. As the LCFBG is one of the key elements of this sensing interrogation system, the characteristics of the LCFBG, such like time delay characteristics and bandwidth should be carefully designed and optimized, e.g., the ripple in time delay response of the LCFBG limits the minimum detectable wavelength change, and also the chirp rate of the LCFBG can also affect the system responsive factor, and so on.
3. The sensing responsive factor of this system can be tailored by designing the chirp rate of the LCFBG or the frequency of the modulated RF signals.

**Figure 18.**
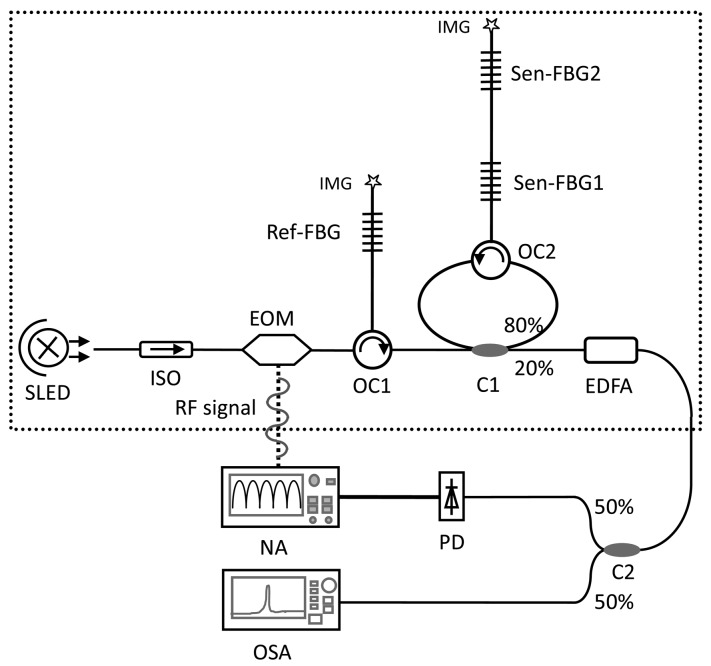
The FBG sensor interrogation system. ISO: isolator, IMG: index-matching glue; superluminescence diode: SLED; electro-optic modulator: EOM; coupler: C1, C2; optical circulator: OC2; sensing FBGs: Sen-FBG1, Sen-FBG2; erbium-doped fiber amplifier: EDFA; optical spectrum analyzer: OSA; photo-detector: PD; network analyzer: NA.

The sensing interrogator by utilizing the Sagnac loop based microwave photonic filter can do real time sensing de-modulation and it's more compact than the dispersive fiber based FBG sensing interrogator; however, it suffers from certain disadvantages, such as the difficulty of multiplexing since the LCFBG has a very broad reflection band, and if numbers of sensing FBGs work at the same time, it's hard to discriminate the power of the recovered RF signal with one frequency from another. In this case, for ease of multiplexing we can use another kind of microwave photonic filter, in which a fiber ring is utilized to form a microwave photonic notch filter with sensing FBGs [70]. Using an additional FBG as a reference, the RF signal after filtering can be utilized to infer the wavelength shift of each sensing FBG. The schematic diagram of this FBG sensor module is shown in Figure 18.

In this schematic diagram, the output at port-3 of C1 is a typical response of microwave photonic notch filter and its frequency response can be expressed as [71]:

(5) $$P_{\text{out}}=P_{0}R_{\text{ref}}(\lambda )\left|A+{\left(1-A\right)}^{2}\sum_{1}^{\infty }{\left(AH\right)}^{k-1}\right|$$

where:

(6) $$H=(1-a)\sum_{i=1}^{2}R_{\text{sen}-i}(\lambda )e^{-j2\pi \;fnL_{i}/c}$$

and *P*_0_ is the output optical power of the SLED, *A* = 0.2 is the couple ratio to port-3 when the light is input from port-1 of the coupler, *a* is the total loss of the light within one round-trip of the fiber ring. *R_ref_*(*λ*)and *R_sen_*_–i_(*λ*) are the reflectivity of the reference FBG and the *i*-th sensing FBG, respectively; *n* is the effective refractive index of the core mode of the fiber, and *f* is the driven frequency of the RF signal onto the EOM; *I_i_* is the round trip length of the fiber ring corresponding to the *i*-th sensing FBG, and *c* is the velocity of light in vacuum.

**Figure 19.**
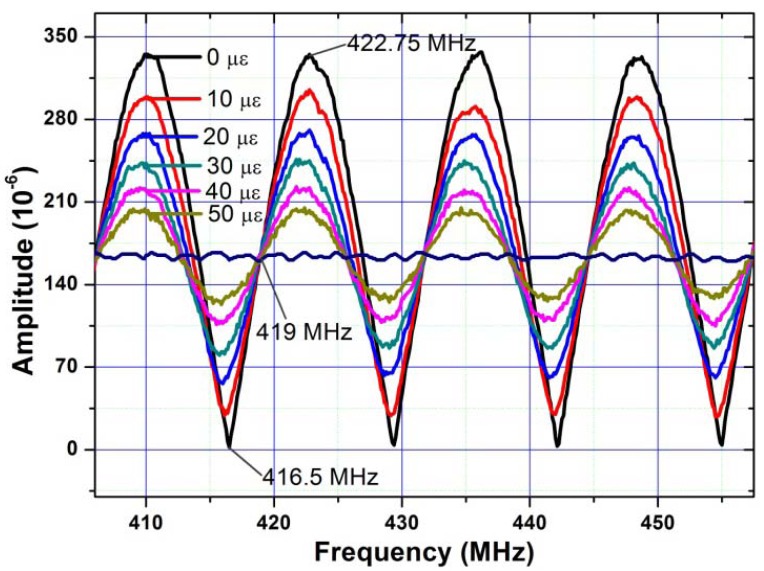
The measured responses of the sensor module when different strains are applied.

When the wavelength of the reference FBG is tuned to match that of FBG1, an RF signal frequency response with FSR1is observed. When the wavelength of the reference FBG is tuned to match that of FBG2, an RF signal frequency response with FSR2 is observed. The axial elongation is applied onto FBG1 with each step of 10 με in the experimental demonstration. The measured RF signal amplitudes at different frequencies with the applied strain are shown in Figure 19, which shows that, the amplitude of the RF signal at the dip and peak frequencies of the frequency response changes quickly with the applied strain.

**Figure 20.**
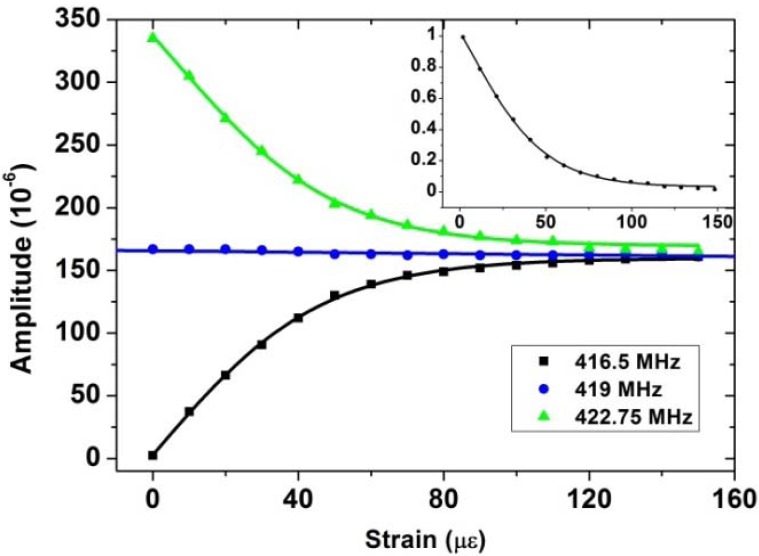
The measured amplitudes of the RF signal as the applied strain varies. The inset shows the normalized peak-dip intensity difference of the output RF signal power as the applied strain varies.

The measured RF signal amplitudes changes with the applied strain at 416.5 MHz (a dip frequency), 419 MHz and 422.75 MHz (a peak frequency) are shown in Figure 20. The slopes of the amplitude changes at dip and peak frequencies for the first 10 με of the applied strain are 3.5 μ/με and 3.2 μ/με, respectively. A small change (less than 0.1 με) in strain can be detected. One can utilize the normalized peak-dip amplitude difference expressed in Equation (6) to measure the strain of the sensing FBG:

(7) $$P_{\text{Nor}}=\frac{P_{422.75\text{MHz}}-P_{416.5\text{MHz}}}{P_{422.75\text{MHz}}+P_{416.5\text{MHz}}}$$

By measuring this normalized peak-dip amplitude difference, the influence of the power fluctuation of the SLED can be eliminated. Strain at another FBG can be monitored by matching the wavelength of the tunable reference FBG and measuring the RF signal with a different FSR in a similar way. Further more, for a multiplexed sensing system composed of numbers of sensing FBGs, RF signal with different frequencies can be modulated onto the light wave and reflected from one of the reference FBG. By tuning the wavelength of the reference FBG to match with different sensing FBGs, with which a microwave photonic filter with different FSR (due to the different time delay) can be achieved; thus RF signals with different frequency can be filtered out, which can be utilized as an identification for different sensing FBGs.

### 3.4. Microwave Generation as a Transversal Loading Sensor

**Figure 21.**
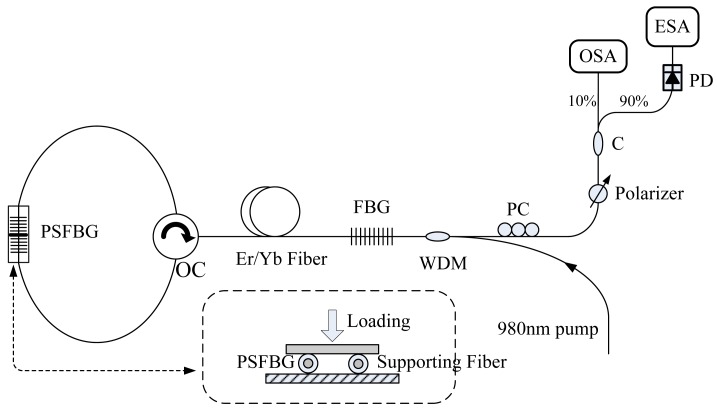
The schematic diagram of the PSFBG based dual-wavelength laser and the microwave generation; inset is the transversal loading configuration. Optical circulator: OC; polarization controller: PC; coupler C; photo-detector: PD; electrical spectrum analyzer: ESA.

Fiber Bragg gratings have been widely used for the photonic generation of microwaves. For example, the dual-phase-shift grating [18,19] and fiber Fabry-Perot interferometer [31] formed by two FBGs have been utilized to achieve a single-longitudinal-mode (SLM) dual-wavelength laser, which can beat to generate a microwave signal at certain frequency. For all these implementations, specially designed FBGs with two sharp transmission peaks are needed to achieve the SLM dual-wavelength lasers. To simplify the fabrication complexness, the tunable microwave generation with a normal phase-shifted FBG (PSFBG, one ultra-narrow transmission peak in the reflection band) has been proposed. Two split transmission peaks can be achieved by applying transversal load on the whole PSFBG part, and thus a SLM dual-wavelength laser can be formed by utilizing it as a wavelength selective element. With different transversal loading, the wavelength spacing of the split peaks will be different. The schematic diagram is shown in Figure 21.

**Figure 22.**
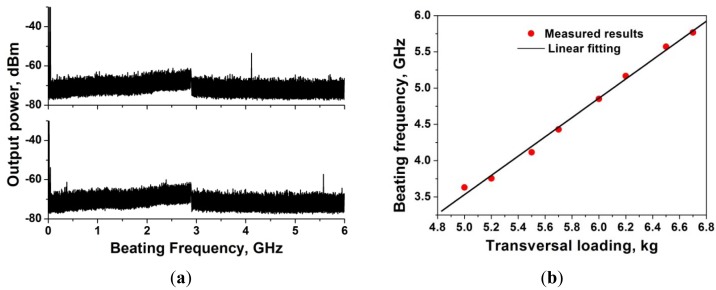
(**a**) The spectra of the beating generated signals when the PSFBG is under different transversal loading and (**b**) the relationship between the value of transversal loading and the frequency of the beating-generated microwave signal.

By beating the two wavelengths, microwave signals will be generated and when different values of transversal loading are applied on the PSFBG, a dual-wavelength laser with different wavelength spacing can be achieved and then beating microwave signals with different frequency will be generated. On the other hand, this scheme can also be used as a transversal loading sensor by tracking the frequency of the microwave signal output. Figure 22(a) shows two spectra of the beating generated signals when the PSFBG is under different transversal loading (above: 5.5 kg; below: 6.5kg), from which one can see that the frequency of the beating microwave signal increases when we increase the load. The measured relationship between the value of transversal loading and the frequency of the beating-generated microwave signal is depicted in Figure 22(b), which shows a good tuning linearity, and that this scheme has the potential as a transversal loading sensor.

In this configuration, the orientation of the applied transversal loading on the PSFBG is an important factor for the beat frequency of the microwave signals, *i.e.*, the frequency of the beating-generated microwave signal can be different, even the same value of loading is applied, only because the loading applied to the different orientation of PSFBG, so the sensitivity of this sensor can be tailored for different usages. Furthermore, since the beating frequency of this scheme only relies on the wavelength spacing of two narrow transmission peaks of the PSFBG with applied different transversal loading, therefore it is insensitive to the temperature.

## 4. Conclusions

In this paper, we have reviewed the applications of advanced photonic technologies, such as fiber lasers and microwave photonic technology in fiber sensing network. Fiber laser based FBG interrogation and the application of Fourier domain mode locking fiber laser for sensing interrogator and sensing network have been described, and for microwave photonic technology, both the applications of microwave photonic filter for FBG sensing interrogation and microwave signal generation used as a transversal loading sensor have been reviewed. The characteristics and sensing performance of these technologies have theoretically analyzed and experimentally demonstrated. Besides the inherent advantages exhibited by fiber technology, such as compact size, light weight, immunity to EMI, and the ease of multiplexing and remote sensing, the sensing schemes based on fiber laser and microwave photonic technologies enjoy higher sensitivity and responsivity than the traditional passive sensing schemes, and by using fiber laser for sensing, the spectra of narrow fiber lasers enables higher sensitivity and resolution; and for microwave photonics technologies, since the small changes in the optical domain correspond to a very big change in RF domain, the sensitivity will be increased dramatically. Also, the high power of fiber laser will increase the measurement accuracy, and as the RF signal processing technology is maturing these days, it's more cost-effective to implement the microwave photonic technology-based sensing and interrogation systems than the fiber laser-based sensing systems. Furthermore, the microwave photonic technology-based sensing techniques show good potential for future wireless and wireline integrated sensing networks. These active sensing techniques show better sensing performance such as higher sensitivities, ease of multiplexing, better responsivities, and the capability for remote sensing and so on. With the development of the advanced photonic technology, more and more novel fiber technologies can be applied in fiber-optic sensing network applications, which can be very beneficial for achieving superior sensing performance for industrial applications and people's everyday lives.
